# Clinical significance of the detection of serum IgG4 and IgG4/IgG ratio in patients with thyroid-associated ophthalmopathy

**DOI:** 10.1515/biol-2022-0694

**Published:** 2023-08-31

**Authors:** JianGuo Zhao, Yajing Ge, Wenjing Li, Zipei Jiang

**Affiliations:** Ophthalmology Department, The First Affiliated Hospital of Wenzhou Medical University, Wenzhou 325000, China

**Keywords:** thyroid-associated ophthalmopathy, immunoglobulin G4, immunoglobulin-G4-related disease

## Abstract

To evaluate the clinical significance of detecting serum IgG4 and the IgG4/IgG ratio in patients with thyroid-associated ophthalmopathy (TAO) and to explore whether high serum IgG4 levels and the IgG4/IgG ratio are associated with the severity and activity of TAO, we retrospectively assessed the records of 78 TAO patients and 32 controls collected in our hospital from July 2020 to July 2022. The TAO patients were divided into TAO inactive and TAO active phase groups according to the clinical activity score (CAS), and we evaluated the association between the serum IgG4 levels, the IgG4/IgG ratio, and the clinical data of the participants. The levels of IgG4 significantly increased in the TAO active group compared to those in the inactive and control groups (*P* < 0.05). Additionally, the number of patients with increased IgG4 levels (≥135 mg/dL) in the TAO active group was markedly higher than that in the inactive and control groups (*P* < 0.05). The IgG4/IgG ratio was also significantly enhanced in the TAO active group compared to the inactive and control groups (*P* < 0.05). CAS was identified as an independent factor influencing IgG4 levels in patients with TAO. The levels of serum IgG4, as well as the IgG4/IgG ratio, were significantly increased in some patients with active TAO, and they were related to the CAS, suggesting that the pathogenesis of TAO may be heterogeneous.

## 1 Introduction

Thyroid-associated ophthalmopathy (TAO) is an organ-specific chronic autoimmune disease closely related to various types of autoimmune thyroiditis (such as Graves’ disease and Hashimoto’s thyroiditis). It can occur in patients with distinctive thyroid functions [1]. TAO is more prevalent in women, with a prevalence rate of approximately 2.5–6% higher than in men [2]. According to some epidemiological surveys, hyperthyroidism is on the rise with the increase in iodine content in salt, and hyperthyroidism is the most significant factor causing TAO [3]. Although many studies have been carried out on the pathogenic factors of TAO, the pathogenesis of TAO still needs to be determined. In recent years, with further study of its etiology and precise pathogenesis, it has been found that a variety of cytokines are involved in the occurrence and development of TAO. It is a consensus that TAO is an organ-specific autoimmune inflammatory disease involving cellular and humoral immunity and other factors. Hence, it is of great significance to study the mechanism of immune inflammation during TAO [4].

Immunoglobulin-G4-related disease (IgG4-RD) is a rare immune-mediated chronic inflammatory disease with fibrosis that can affect various organ systems [5]. IgG4-RD has complex and varying clinical presentations. The typical clinical manifestations of IgG4-RD are organ involvement and increased serum IgG4 levels [6]. Initially found to be associated with autoimmune pancreatitis, IgG4-RD has been recently reported to be related to other organs, including the kidney, bile ducts, parotid gland, and so on [7,8]. Recently, it has been suggested that IgG levels are altered in autoimmune diseases [9,10]. Recently, it has been reported that orbital diseases are related to IgG4. However, the relationship between IgG4 and TAO’s severity and activity is unclear. Although some studies suggest a positive correlation between IgG4 levels and the development of TAO, these investigations may draw incomplete conclusions due to their limitations, such as only including patients with a high clinical activity score (CAS) (≥4) or patients with severe TAO [11,12]. We hypothesize that serum IgG4 levels and the IgG4/IgG ratio may serve as predictors for the progression of TAO. Therefore, this case–control study was carried out to explore the distributional characteristics of serum IgG4 in patients with TAO in different stages and the predictive value of serum IgG4 and IgG4/IgG ratio in identifying whether TAO would enter the active stage, guiding clinical practice.

## 2 Methods

### 2.1 Participants

In this retrospective case–control study, we analyzed TAO patients who had undergone serum IgG subclass examination at our hospital between July 2020 and July 2022 by consulting medical records. We collected the following details: gender, age, type of thyroid disease, TAO course, exophthalmos, CAS, TAO severity, serum IgG4, and IgG levels of all selected patients. The serological examination results of the control population hospitalized during the same period were used as the control group. This study was approved by the Ethics Committee of the first affiliated hospital of Wenzhou medical university.


**Informed consent:** Informed consent has been obtained from all individuals included in this study.
**Ethical approval:** The research related to human use has been complied with all the relevant national regulations, institutional policies and in accordance with the tenets of the Helsinki Declaration, and has been approved by Ethics Committee of the first affiliated hospital of Wenzhou medical university.

### 2.2 Inclusion and exclusion criteria

The TAO diagnosis was based on the Bartley criteria [13]. TAO patients with normal thyroid function and hypothyroidism were excluded. Patients with local or systemic immune or inflammatory paralysis were excluded. Patients who had used glucocorticoids or immunosuppressants in the past 3 months and pregnant or lactating women were also excluded.

### 2.3 Research methods

#### 2.3.1 Measurement of exophthalmos

The Hertel bulbar protrusion meter was used to measure the vertical distance from the orbit’s outer edge to the cornea’s apex. The exocytosis of both eyes was measured, and the average value was analyzed.

#### 2.3.2 Course of TAO and CAS

The interval between the first occurrence of ocular symptoms and hospitalization was calculated as the course of TAO, according to the standard of Mourits et al. [14]. The standard score range for CAS was 0–10, where ≥3 was classified as the active stage and <3 as the inactive stage [14].

#### 2.3.3 TAO range grading

According to Bartalena’s standards, the range is graded as light, moderate, and severe. The mild category meets the following four conditions: (1) no diplopia or intermittent diplopia, (2) mild soft tissue swelling, (3) increased protrusion of the eyeball <3 mm, and (4) retraction of the eyelid <2 mm. Moderate category meets any of the following four conditions: (1) diplopia occurs in the first eye position, (2) moderate soft tissue swelling, (3) increased ocular protrusion ≥3 mm, and (4) eyelid retraction ≥2. Severity is defined as the presence of corneal ulcer or optic nerve involvement.

#### 2.3.4 Detection of serum immunoglobulin

Venous blood was collected from patients. Immune scattering turbidimetry was used to measure the levels of serum IgG4 and IgG subtypes. A fully automatic turbidimeter (Beckman AU5800) was used as the testing instrument.

### 2.4 Statistical analyses

The continuous variables were presented as mean ± standard deviation or median (quartile 25–75). Categorical variables were compared using the *χ* ^2^ test. The differences in continuous variables among the groups were evaluated by the one-way ANOVA or Kruskal–Wallis test. All statistical analyses were carried out with SPSS 25 software (IBM Corp). *P*  <  0.05 was considered statistically significant.

## 3 Results

### 3.1 Demographic characteristics of participants

**Figure 1:**
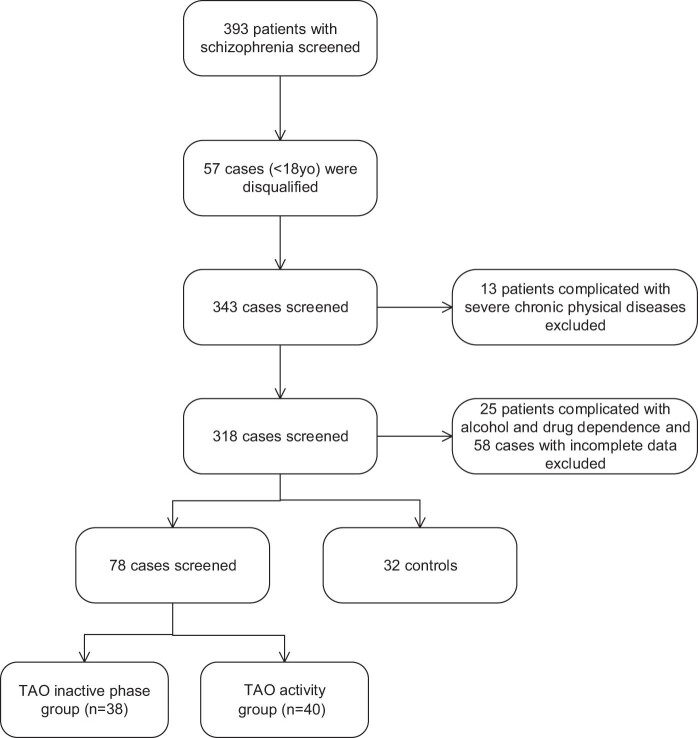
The graphical abstract for this study.

The initial exploration of our hospital’s electronic medical system revealed 176 patients with TAO from July 2020 to July 2022. Fifteen cases under the age of 18 were disqualified. After excluding 23 patients with severe chronic physical illnesses, 12 patients with alcohol and drug dependence, and 48 cases with incomplete data, 78 patients with TAO and 32 controls were qualified for this study (Figure 1). According to the CAS, TAO patients were divided into groups based on the active and inactive phases. The gender of patients, age, course of TAO, degree of exophthalmos, and severity of TAO and CAS are demonstrated in Table 1. No significant difference was observed in gender and age among the three groups (all *P* > 0.05). The degree of exophthalmos and CAS in the TAO active group was significantly higher than in the inactive group (all *P* < 0.05). The disease duration was significantly shorter in the TAO active group than in the inactive group (*P* < 0.05).

**Table 1:** Clinical data of TAO active group, TAO inactive group, and control group

	Control group	TAO inactive phase group	TAO activity group	*P* value
Cases	32	38	40	
Gender				0.923^&^
Male	8 (25%)	9 (23.68%)	9 (22.5%)	
Female	24 (75%)	29 (76.32%)	31 (77.5%)	
Age (years)	40.63 ± 8.12	36.5 ± 12.56	43.11 ± 8.02	0.071^Φ^
TAO course (months)	—	46.83 ± 48.35	18.56 ± 14.63	0.008*^Φ^
Degree of exophthalmos (mm)	—	17.77 ± 2.43	21.14 ± 3.58	0.000*^Φ^
CAS	—	0.83 ± 0.84	4.47 ± 0.81	0.000*^#^
TAO severity degree	—			0.000^&^
Light	—	7 (18.42%)	0	
Medium	—	31 (81.58%)	8 (20%)	
Serious	—	0	32 (80%)	

Data were presented as mean ± standard deviation or number (%). *P*-values were obtained using ^&^χ2 tests for categorical variables; the ^#^Kruskal–Wallis test or ^Φ^one-way ANOVA were used for continuous variables. The asterisk implies statistical significance.

### 3.2 Serum IgG4 and immunoglobulin levels among the active phase group, inactive phase group, and control group

**Figure 2:**
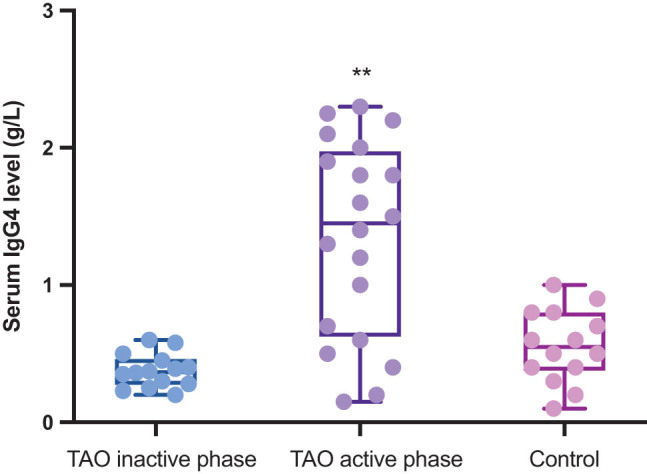
Comparison of serum IgG4 levels among the active, inactive, and control groups. **P < 0.05 TAO activity phase group vs TAO inactivity phase group.

The levels of serum IgG4, IgG, and other immunoglobulins in the serum were evaluated among the three groups. The results demonstrated that serum IgG4 levels were markedly higher in the TAO activity phase group than in the TAO inactive phase and control groups (*P* < 0.05, Table 2 and Figure 2). The IgG4/IgG ratio of the TAO activity group was significantly higher than that of the inactive and control groups (*P* < 0.05, Table 2 and Figure 1). The percentage of patients with serum IgG4 levels higher than 135 mg/L in the TAO inactive, TAO active, and control groups were 5.26% (2/38), 27.5% (11/40), and 6.25% (2/32), respectively.

**Table 2:** Comparison of serum IgG4 and immunoglobulin levels among active, inactive, and control groups

	Control group	TAO inactive phase group	TAO activity group	*P* value
Cases	32	38	40	
IgG4 (g/L)	0.24 (0.15, 0.36)	0.32 (0.14, 0.53)	0.55 (0.24, 0.67)	0.041^Φ^*
IgG4/IgG (%)	2.12 (1.67, 6.88)	2.80 (1.58, 5,79)	5.85 (2.83, 6.89)	0.006^#^*
IgE (IU/mL）	67.09 (36.77, 75.98)	110.05 (72.00, 140)	130.44 (69.084, 150.77)	0.902^Φ^
IgA (g/L)	3.80 (2.49, 13.55)	2.21 (1.69, 3.48)	2.45 (1.59, 6.05)	0.880^Φ^
Percentage of patients with serum IgG4 >135 mg/L	2 (6.25%)	2 (5.26%)	11 (27.5%)	0.07^&^
Complement C3 (g/L)	1.47 (0.78, 1.79)	1.33 (0.57, 2.39)	1.20 (0.58, 4.90)	0.670^#^
Complement C4 (g/L)	0.34 (0.13, 0.67)	0.27 (0.17, 0.58)	0.25 (0.10, 0.84)	0.070^#^

Data were presented as medians with interquartile ranges or numbers (%). *P*-values were obtained using ^&^χ2 tests for categorical variables; the ^#^Kruskal–Wallis Test or ^Φ^one-way ANOVA were used for continuous variables. The asterisk implies statistical significance.

### 3.3 Comparison of a positive rate of serum IgG subtypes in different groups

Compared to the control group, the favorable rates of serum IgG1 and IgG4 in the TAO inactive phase and TAO activity phase groups were higher than those in the control group (*P* < 0.05, Table 3). There was no significant difference in the positive rate of serum IgG2 and IgG3 among the different groups (*P* > 0.05).

**Table 3:** Comparison of positive rate of IgG subtypes in different groups

	Control group	TAO inactive phase group	TAO activity group	*P* value
Cases	32	38	40	
IgG1 (g/L)	6 (18.75%)	23 (60.53%)	29 (72.50%)	0.001*
IgG2 (g/L)	0 (0)	4 (10.53%)	5 (12.50%)	0.706
IgG3 (g/L)	5 (15.6%)	11 (28.95%)	13 (32.50%)	0.082
IgG4 (g/L)	3 (9.37%)	20 (52.63%)	21 (52.50%)	0.000*

Data were presented as numbers (%). *P*-values were obtained using *χ* ^2^ tests for categorical variables. The asterisk implies statistical significance.

### 3.4 Association between CAS score and serum IgG4 levels

**Figure 3:**
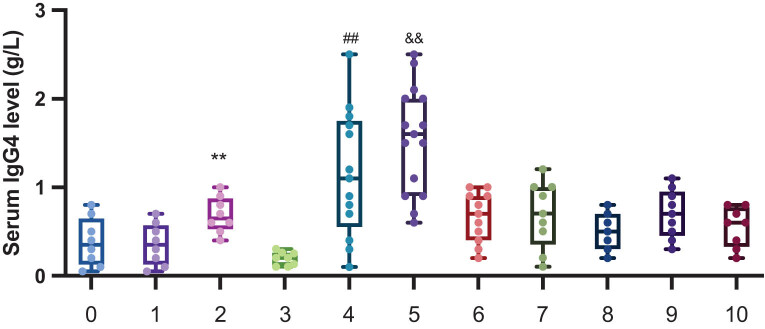
Serum lgG4 levels in TAO patients with different CAS groups. **P < 0.05 CAS2 vs CAS0. ^##^ *P* < 0.01 CAS4 vs CAS3. ^&&^ *P* < 0.001 CAS6 vs CAS5.

This study divided TAO patients into seven groups, according to the CAS. The serum IgG4 levels of the seven groups are shown in Table 4 and Figure 3. The results suggest that the CAS is an independent influencing factor of serum IgG4 (*P* < 0.05) (Figure 2).

**Table 4:** CAS score and serum IgG4 levels in patients with TAO

CAS	Cases	lgG4 (g/L)		
		Median	*Q*1 (25% Quantile)	*Q*3 (75% Quantile)
0	15	0.35	0.17	0.65
1	16	0.26	0.12	0.53
2	6	0.61	0.3	0.72
3	2	0.23	0.17	0.32
4	20	0.72	0.25	2.04
5	11	0.55	0.19	2.63
6	8	0.16	0.09	0.33
*x* ^2^	13.618			
*P*	0.02*			

*P*-values were obtained using *χ*2 tests for categorical variables. The asterisk implies statistical significance.

### 3.5 Serum IgG4 levels in patients with a different severity content of TAO

The TAO patients in this study were classified according to the severity degree and divided into three groups: mild (*n* = 7), moderate (*n* = 38), and severe (*n* = 33). The corresponding serum IgG4 levels are shown in Table 5. There is no significant difference in serum IgG4 levels among the severity groups (*P* > 0.05).

**Table 5:** Serum IgG4 levels in TAO patients with varying degrees of severity

TAO severity degree	Cases	lgG4 (g/L)		
		Median	*Q*1 (25% Quantile)	*Q*3 (75% Quantile)
Mile	7	0.45	0.2	0.69
Moderate	38	0.33	0.22	0.58
Severe	33	0.712	0.41	0.77
*x* ^2^	3.311			
*P*	0.187			

*P*-values were obtained using *χ* ^2^ tests for categorical variables.

## 4 Discussion

TAO is an autoimmune disease in which helper T cells play a leading role mediated by autoantibodies [15]. The occurrence of orbital IgG4-related diseases is also related to the activation of the immune system by helper T cells in the body, resulting in abnormal serum and immune function. Therefore, some scholars believe that there is a correlation between TAO and orbital IgG4-related diseases [16]. In addition, it has been reported that serum IgG4 levels are significantly increased in patients with Hashimoto’s thyroiditis and Graves’ disease [17,18]. A recent study has suggested that after treatment with high-dose glucocorticoid, the related symptoms of patients with Graves’ ophthalmopathy were significantly improved, and the level of serum IgG4 decreased [19]. In addition, an increasing number of studies have suggested that patients with Graves’ ophthalmopathy with high levels of serum IgG4 seem to achieve a better curative effect, while patients with Graves’ ophthalmopathy with low levels of serum IgG4 are less effective than the former, suggesting that IgG4 may be used as a target to affect the therapeutic effect of glucocorticoid [20]. In this study, we speculated that the level of serum IgG4 in patients with different TAO might be changed and be associated with the severity and activity of TAO.

IgG contains four subtypes: IgG1, IgG2, IgG3, and IgG4, of which the content of IgG1 is the highest and the content of IgG4 is the lowest [21]. IgG4 in the serum of healthy people accounts for only 3–6% of the total IgG [22]. Olejarz et al. suggested that 6.4% of patients with Graves’ disease have serum IgG4 ≥135 mg/dL [19]. Nagata et al. demonstrated that 9.09% of patients with Graves have serum IgG4 ≥135 mg/dL [23]. In this study, we found that the percentage of patients with serum IgG4 levels higher than 135 mg/L in the TAO inactive phase, TAO active phase, and control groups were 5.26% (2/38), 27.5% (11/40), and 6.25% (2/32), respectively. The elevated levels of serum IgG4 and IgG4/IgG in some TAO patients suggest that there might be heterogeneity in the pathogenesis of TAO, as with Hashimoto’s thyroiditis and Graves’ disease. The pathogenesis of TAO is not entirely clear. However, some studies have shown that as an organ-specific autoimmune inflammatory disease, TAO’s pathogenesis involves cellular immunity, humoral immunity, and other factors [24]. Other groups have previously reported that antigen-specific and antigen-independent pathways play essential roles in the pathogenesis of TAO. The increasing comprehension of the immunopathogenesis of TAO is leading to the application of new therapies that have exposed particular potential in patients resistant to conventional therapies. As the experience of these treatments increases, further research to assess short-term and long-term efficacy and potential adverse reactions will be crucial.

As a particular serological marker that can be present in various autoimmune diseases, the relationship between serum IgG4 and TAO deserves more research and attention. Evidence has indicated that serum IgG4 to IgG4/IgG increased in some patients with TAO [25]. Recently, similar studies underscored that in moderate to severe TAO patients undergoing orbital decompression or those threatening vision (a complicated situation), the positive rate of serum IgG4 is up to 20%, and the favorable ratio of serum IgG4 in TAO patients who threaten vision can be as high as 25.3% [26]. In addition, Gonzalez-Diaz et al. found that the level of serum IgC4 in patients with Graves ophthalmopathy was significantly higher than in patients with Graves’ disease without ophthalmopathy and normal controls [27]. The study of Gonzalez-Diaz et al. has some limitations. In their study, patients with Graves ophthalmopathy were not grouped according to disease activity. In our study, we divided TAO patients into inactive and active phase groups according to the CAS, effectively solving the limitation of Gonzalez-Diaz’s research. The results of our study demonstrated that the level of serum IgG4 in patients with active TAO was significantly higher than that in patients with inactive TAO and control groups. Moreover, Bozkirli et al. suggested a linear correlation between CAS and IgG4 [28]. They believe that serum IgG4 may be one of the factors in screening Graves’ ophthalmopathy risk and choosing the proper treatment for Graves’ ophthalmopathy. However, Bozkirli’s study had limitations as they only enrolled 37 patients with Graves’ ophthalmopathy. The population was relatively small in a single university. There may be differences between our results and Bozkirli’s, which might be due to the following factors: (1) heredity and genes are essential factors in the pathogenesis of TAO, and different ethnic groups in the two studies may lead to differences in results. (2) The natural course of TAO in the active phase is generally 18–36 months, and then it gradually enters the inactive stage. Our findings validated that the serum IgG4 level in active TAO patients was significantly higher than that in the inactive stage, suggesting the role of IgG4 in the early pathogenesis of TAO.

The diagnosis and evaluation of TAO currently rely mainly on clinical manifestations and imaging examinations. CAS is a qualitative judgment based on a series of clinical signs, which is a crucial indicator to evaluate the activity of TAO and whether to carry out anti-inflammatory treatment orbital radiotherapy [29]. There is a need for more early, accurate, sensitive, and convenient observation indicators. It has been reported that TSH-binding inhibitory immunoglobulin and thyroid-stimulating immunoglobulin are closely related to the pathogenesis and persistence of TAO [30]. After grouping TAO patients according to CAS in this study, the number of patients in the subgroups is small. Due to limitations in conditions, this study only observed 78 TAO patients, and the sample size needs to be more significant. In this experiment, because each subtype belongs to a different detection system, it has different types and concentrations of enzyme-labeled secondary antibodies, which limits the comparison between different subtypes. Therefore, in our further investigation, we plan to expand the number of cases, improve the statistical test’s efficiency, further clarify the exact relationship between CAS and serum IgG levels, compare the changing trend of serum IgG levels in TAO patients before and after treatment, and analyze the relationship between serum IgG4 levels and treatment response and clinical outcomes. These efforts are aimed at improving the predictability of clinical diagnosis and treatment.

To sum up, this study found that serum IgG4 and IgG4/IgG levels were significantly increased in patients with active TAO but did not belong to the IgG4-related disease. The level of serum IgG4 in patients with TAO is related to CAS, which suggests that IgG4 may be involved in part of the pathological mechanism of TAO. Determining serum IgG4 level helps manage TAO and choose the appropriate treatment regimen. High IgG4 levels may help to screen out patients with high-risk factors of TAO in GD patients. Given the diagnostic and staging value of orbital MRI in TAO, combined with MRI examination is helpful to clarify the value of serum IgG4 in the prediction, diagnosis, and treatment of TAO, improve the predictability of clinical diagnosis and treatment, and provide new ideas for clinical diagnosis and treatment of TAO.
